# Novel amphiphilic graft block azobenzene-containing copolymer with polypeptide block: synthesis, self-assembly and photo-responsive behavior†

**DOI:** 10.1039/c9ra10351a

**Published:** 2020-02-05

**Authors:** Xiaohua He, Jianxiang Wu, Chunyan Gao

**Affiliations:** School of Chemistry and Molecular Engineering, East China Normal University 500 Dongchuan Road Shanghai 200241 China xhhe@chem.ecnu.edu.cn

## Abstract

Well-defined amphiphilic graft block azobenzene-containing copolymer with polypeptide block was synthesized *via* a combination of copper-mediated atom transfer radical polymerization (ATRP), ring-opening polymerization and click reaction. The alkyne-terminated poly[6-(4-methoxy-azobenzene-4′-oxy)hexyl methacrylate] (PAzoMA) was synthesized by ATRP with a bromine-containing alkyne bifunctional initiator, and the azido-terminated poly(γ-2-chloroethyl-l-glutamate) (PCELG) was synthesized by ROP of γ-2-chloroethyl-l-glutamate-*N*-carboxyanhydride (CELG-NCA), then the two homopolymers were conjugated by click reaction to afford block azobenzene-containing copolymer PAzoMA-*b*-PCELG. The chloro groups in PCELG block were transformed into azido groups *via* azide reactions, and the alkyne-terminated MPEG was grafted to the polypeptide block to afford the final product PAzoMA-*b*-poly((l-glutamate)-*graft*-methoxy poly(ethylene glycol)) (PAzoMA-*b*-(PELG-*g*-MPEG)) by click reaction. Giant vesicles (micrometer size) were obtained from the amphiphilic graft block copolymer PAzoMA-*b*-(PELG-*g*-MPEG) through a solution self-assembly due to the rigid PAzoMA chains and polypeptide chains with the α-helical structure. The investigation of the photo-isomerization behavior of PAzoMA-*b*-(PELG-*g*-MPEG) in solution and in vesicular solution showed *trans*–*cis* isomerization in solution was quicker than that in vesicular solution and azobenzene J-aggregates in the vesicle solution were only observed. The formation mechanisms of the vesicles were also explored. The research results may provide guidelines for the study of complex copolymers containing different types of rigid chains.

## Abstract 2

Giant vesicles (micrometer size) were prepared from novel amphiphilic graft block azobenzene-containing copolymer with polypeptide block synthesized *via* a combination of ATRP, ROP and click reaction.

## Graphical abstract

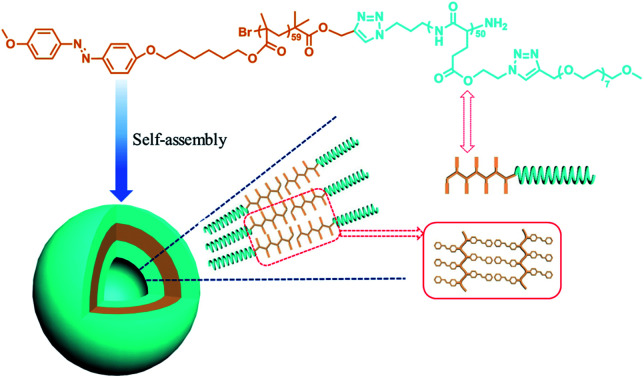

## Introduction

Recently, azobenzene-containing polymers (*i.e.* azo-polymers) have attracted much attention because they exhibit unique photo-responsive characteristics, such as photoisomerization, photochemical phase transitions, photoalignment and so on.^1–4^ Their photo-responsive behavior depend not only on the chemical structures of azobenzene chromophore groups, but also on their organization in the polymer matrix. To deeply explore their photo-responsive behavior, azobenzene-containing copolymers with different architectures, such as random copolymers, block copolymers, star-like copolymers, cyclic diblock copolymers and dendritic copolymers and so on, have been designed and synthesized.^4–30^ For instance, del Barrio and Oriol *et al.* prepared a series of azobenzene-containing linear-dendritic copolymers by using click chemistry.^15^ Li and Zhu *et al.* reported the preparation of cyclic azobenzene-containing amphiphilic diblock copolymers *via* the reversible addition-fragmentation chain transfer (RAFT) polymerization.^29^ Our research group also reported the preparation of azobenzene-containing copolymers with different architectures including AB, ABC and ABC_2_ type block copolymers and ABC type star copolymers by RAFT, ATRP and click reaction.^4,7,26–28^ The self-assembly behavior of these synthetic azobenzene-containing copolymers have usually been studied in detail. The reasons may be that azobenzene-containing polymers are used as model rod-like polymers due to the stronger π–π stacking interaction within azobenzene structures, which is helpful to understand deeply the self-assembly behavior of rod-like block copolymers.^31,32^ On the other hand, the photoisomerization of azobenzene structures is also affected by the microenvironments in which azobenzenes are located.^2,7,8,26,33–35^ Li *et al.* found that azobenzene-containing linear-dendritic diblock copolymers can be assembled into cylindrical micelles, sheet-like micelles, tubular micelles, and polymer vesicles by tubing the generations of azobenzene-containing dendrons in linear-dendritic copolymers.^9^ Our research group reported the self-assembly behavior of azobenzene-containing copolymers and different self-assembled structures, such as spherical micelles, bowl-shaped micelles and large compound vesicles and so on, have been obtained by tailoring their chemical structures and the solution concentrations.^25,26,36,37^ Meanwhile, these formed aggregates can be deformed under irradiation with polarized UV light, which is due to the photoisomerization of azobenzene structures.^25,36,37^ These studies deepen the understanding of azobenzene-containing materials.

A growing research interesting has also been shown in the synthesis of block copolymers comprised of polypeptide segments.^38–46^ Compared with conventional polymers, polypeptides can show well-defined stable secondary conformations including α-helices, β-sheets and random coils, which plays an important role in the formation of the aggregated structures in block polypeptides. Especially speaking, polypeptides can also serve as model rod-like polymers in solution and in the solid state because of their rigid α-helical conformation structures formed by intramolecular hydrogen bonding interactions in polypeptide chain.^38,39,47,48^ Deming and co-workers reported the preparation of large-size vesicles from copolypeptides through conformation-specific self-assembly.^40,41^ Lin *et al.* obtained the super-helical aggregate structures and abacus-like structures assembled from a binary system consisting of amphiphilic blocks copolypeptide poly(ethylene glycol)-*block*-poly(γ-benzyl-l-glutamate) (PEG-*b*-PBLG) and homopolypeptide PBLG.^42,43^ Recently, our research group also reported disk-like micelles with cylindrical pores from PEG-*b*-PBLG.^45^ The research results showed that the α-helical conformation of PBLG blocks plays a key role in the formation process of different aggregate structures. On the other hand, polypeptides as rod model polymers are usually introduced into other functional polymer systems and constructed into functional block copolymer systems. For example, Manners *et al.* reported that metallopolymer polyferrocenylsilane (PFS) was introduced into polypeptide systems and constructed into PFS-*b*-polypeptide block copolymers, and studies on their self-organization behavior in both the bulk state and in solution were performed.^49–52^ Recently, our research group prepared azobenzene-containing diblock copolymers with PBLG block by using a combination of ATRP, ROP and click reaction.^7^ These research works can expand the research scope of copolypeptides.

**Scheme 1:**
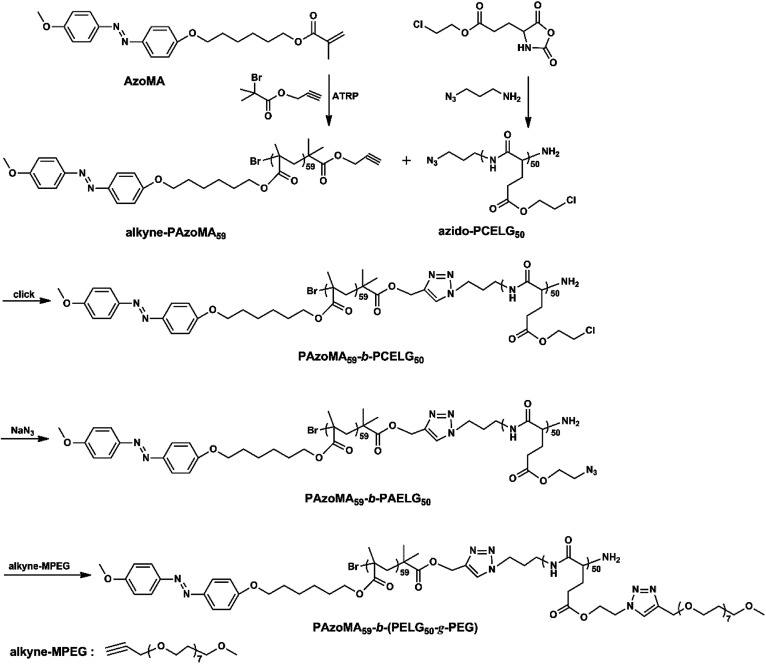
Synthesis of graft block copolymer.

Herein, well-defined amphiphilic graft block azobenzene-containing copolymer with polypeptide block was synthesized *via* a combination of ATRP, ROP and click reaction (Scheme 1). The graft block copolymer consisted of azobenzene-containing poly[6-(4-methoxy-azobenzene-4′-oxy)hexyl methacrylate] (PAzoMA), polypeptide poly(γ-2-chloroethyl-l-glutamate) (PCELG) and hydrophilic poly(ethylene glycol) monomethylether (MPEG), in which the introduction of hydrophobic PAzoMA into PCELG by using click reaction was formed into block copolymer and then hydrophilic MPEG was grafted to the side-groups of polypeptide blocks of the block copolymer by using click reaction. Therefore, the amphiphilic copolymer combined both graft and block structures, PAzoMA-*b*-poly((l-glutamate)-*graft*-methoxy poly(ethylene glycol)) (PAzoMA-*b*-(PELG-*g*-MPEG)), was prepared. In this novel graft block copolymer contained two different arrangement ways of the monomers, two kinds of different rod-like polymer chains derived from different interactions (π–π stacking interaction and intramolecular hydrogen bonding) were constructed into the same system, which may make the copolymer show unique aggregation behaviors. The self-assembled morphologies in selective solvents and photo-responsive behavior of PAzoMA-*b*-(PELG-*g*-MPEG) were investigated. These results may provide guidelines for the design of effective photoresponsive anisotropic materials.

## Experimental

### Materials

6-(4-Methoxy-azobenzene-4′-oxy)hexyl methacrylate (AzoMA) was synthesized according to the procedure reported by Stewart and Imrie.^53^ Propargyl 2-bromoisobutyrate and 1-azido-3-aminopropane were synthesized according to the procedure reported by our group.^7^ γ-2-Chloroethyl-l-glutamate (CELG) and γ-2-chloroethyl-l-glutamate *N*-carboxyanhydride (CELG-NCA) were synthesized according to the literature.^54^ CuBr (Shanghai Chemical Reagent Co., A.R. grade) and chlorobenzene (C_6_H_5_Cl, Acros, 99%) were purified according to the literature.^7^ Polyethylene glycol monomethyl ether (MPEG, *M*_n_ = 350, 99%) and propargylic bromide (99%) were purchased from Aldrich. Trifluoroacetic acid (TFA, A.R. grade), *N*,*N*-dimethylformamide (DMF, A.R. grade) and other solvents were purchased from Shanghai Chemical Reagent Co. and purified by conventional procedures if needed. Sodium azide (NaN_3_, Acros, 99%), l-glutamic acid (Shanghai Chemical Reagent Co., A.R. grade) and other reagents were used as received.

### Synthesis of alkyne-PAzoMA

PAzoMA with alkynyl as a terminal group (alkyne-PAzoMA) was synthesized by using ATRP.^7^ A Schlenk tube was charged with propargyl 2-bromoisobutyrate (28 μL, 0.18 mmol), AzoMA (5.00 g, 12.6 mmol), CuBr (35.0 mg, 0.24 mmol), PMDETA (43.0 mg, 0.24 mmol), C_6_H_5_Cl (8.0 mL) and a magnetic bar. The mixture was degassed by three freeze–pump–thaw cycles, and immersed in a thermostatic coil bath at 85 °C for 12 h under nitrogen. The reaction was stopped and diluted with THF. The copper salts were removed by passing through a column of neutral aluminum oxide, and precipitated into diethyl ether. The product was purified by reprecipitating three times from THF to diethyl ether and dried in a vacuum overnight at 40 °C.

Yield: 80.0%, *M*_n_ (GPC) = 11.0 × 10^3^ kg mol^−1^, *M*_w_/*M*_n_ = 1.26, DP (^1^H NMR) = 59. ^1^H NMR (CDCl_3_, *δ*/ppm): 7.81 (br, –C_6_H_4_–), 6.93 (br, –C_6_H_4_–), 4.61 (br, –CCH_2_–), 3.93 (br, –OCH_2_–), 3.82 (br, CH_3_O–), 1.06 (br, –CCH_2_–), 0.86 (br, CH_3_C–). Thus, the obtained polymer was denoted as alkyne-PAzoMA_59_.

### Synthesis of azido-PCELG

Poly(CELG) with azido group as a terminal group (azido-PCELG) was synthesized by ROP of the CELG-NCA in anhydrous DMF using 1-azido-3-aminopropane according to the literature.^54^ In a typical experiment, CELG-NCA (0.76 g, 3.2 mmol) was added into an already dried two-necked flask with a magnetic bar under pure nitrogen atmosphere. 7.0 mL of anhydrous DMF was introduced into the flask under pure nitrogen atmosphere and the solution was stirred for 15 min, and then 5.1 μL (53.0 μmol) of 1-azido-3-aminopropane was added into the solution using a degassed syringe. After stirring for 3 days in pure nitrogen at room temperature, the solution was concentrated and then precipitated into an excess amount of methanol. The white power product was obtained by filtration and purified by precipitating three times from DMF into methanol, and dried at room temperature in a vacuum oven overnight.

Yield: 81.0%, *M*_n_ (GPC) = 5.2 × 10^3^ kg mol^−1^, *M*_w_/*M*_n_ = 1.29, DP (^1^H NMR) = 50. ^1^H NMR (CDCl_3_ + 15% TFA, *δ*/ppm): 7.88 (br, –NHCO–), 4.66 (br, –CHCO–), 4.37 (t, ClCH_2_CH_2_O–), 3.67 (t, ClCH_2_CH_2_O–), 3.40 (br, N_3_CH_2_–), 2.56 (br, –COCH_2_CH_2_–), 2.20–2.02 (d, –COCH_2_CH_2_–). Thus, the obtained polymer was denoted as azido-PCELG_50_.

### Synthesis of PAzoMA-*b*-PCELG block copolymer by click reaction

The block copolymer PAzoMA-*b*-PCELG was synthesized by click reaction according to the literature.^7,26^ A typical experimental procedure was as follows: alkyne-PAzoMA_59_ (0.70 g, 30.0 μmol, 1.0 equiv.), azido-PCELG_50_ (0.28 g, 30.0 μmol, 1.0 equiv.), CuBr (4.3 mg, 30.0 μmol, 1.0 equiv.) and PMDETA (5.2 mg, 30.0 μmol, 1.0 equiv.) were dissolved in 8 mL DMF, and the mixture was degassed by three freeze–pump–thaw cycles, and then immersed in a thermostatic oil bath at 40 °C for 2 days. The reaction was stopped and the solution was diluted with THF. After removing copper salt through a column of neutral aluminum oxide, the solution was concentrated and precipitated into methanol. The product can be obtained by filtration, purified by reprecipitating three times from THF to methanol and dried at 40 °C under vacuum overnight.

Yield: 90.5%, *M*_n_ (GPC) = 11.2 × 10^3^ kg mol^−1^, *M*_w_/*M*_n_ = 1.25, ^1^H NMR (CDCl_3_ + 15% TFA, *δ*/ppm): 7.97 (br, –C_6_H_4_–), 7.88 (br, –NHCO–), 7.09 (br, –C_6_H_4_–), 4.66 (br, –CHCO–), 4.37 (t, ClCH_2_CH_2_O–), 4.10 (br, –OCH_2_–), 3.97 (br, CH_3_O–), 3.67 (t, ClCH_2_CH_2_O–), 2.56 (br, –COCH_2_CH_2_–), 1.10 (br, –CCH_2_–), 0.92 (br, CH_3_C–). The block copolymer was denoted as PAzoMA_59_-*b*-PCELG_50_.

### Synthesis of PAzoMA-*b*-PAELG block copolymer

The block copolymer PAzoMA-*b*-poly(γ-2-azidoethyl-l-glutamate) (PAzoMA-*b*-PAELG) was synthesized by azide reaction according to the literature.^48^ PAzoMA_59_-*b*-PCELG_50_ (0.64 g, 0.97 mmol of chloro groups) and sodium azide (0.63 g, 9.70 mmol) were dissolved in 8 mL DMF and stirred at 60 °C under nitrogen protection for 48 h. After removing the solvent under vacuum, the residue was extracted with CHCl_3_ three times and the remaining solid was discarded. The CHCl_3_ solution was washed three times with water, concentrated under vacuum and precipitated into diethyl ether. The product was obtained by centrifugation and dried at 30 °C under vacuum overnight.

Yield: 92.6%, *M*_n_ (GPC) = 12.1 × 10^3^ kg mol^−1^, *M*_w_/*M*_n_ = 1.29, ^1^H NMR (CDCl_3_ + 15% TFA, *δ*/ppm): 7.97 (br, –C_6_H_4_–), 7.88 (br, –NHCO–), 7.09 (br, –C_6_H_4_–), 4.66 (br, –CHCO–), 4.30 (t, N_3_CH_2_CH_2_O–), 4.10 (br, –OCH_2_–), 3.97 (br, CH_3_O–), 3.50 (t, N_3_CH_2_CH_2_O–), 2.56 (br, –COCH_2_CH_2_–), 1.10 (br, –CCH_2_–), 0.92 (br, CH_3_C–). The obtained polymer was denoted as PAzoMA_59_-*b*-PAELG_50_.

### Synthesis of alkyne-MPEG

MPEG with alkynyl as a terminal group (alkyne-MPEG) was synthesized by Williamson etherification according to the literature.^55^ MPEG (9.8 g, 28.0 mmol), NaOH (11.4 g, 0.28 mol) and 25 mL toluene were added into a 150 mL round-bottom flask equipped with a magnetic bar. The solution of propargylic bromide (32.5 g, 0.27 mol) in toluene was then added slowly with stirring under N_2_ atmosphere. The reaction mixture was stirred for 15 h at 50 °C. After removing the solvent under vacuum, the residue was extracted with CH_2_Cl_2_ three times and the remaining solid was discarded. The CH_2_Cl_2_ solution was washed three times with water, dried over Na_2_SO_4_ and filtered. The solvent was removed by rotary evaporating and the light yellow viscous liquid was obtained and dried at 30 °C under vacuum overnight.

Yield: 80%, ^1^H NMR (CDCl_3_, *δ*/ppm): 4.16 (s, 2H, CHCCH_2_–), 3.62 (m, 4H, –CH_2_CH_2_–), 3.33 (s, 3H, –CH_3_O–), 2.41 (br, 1H, CH–).

### Synthesis of graft block copolymer PAzoMA-*b*-(PELG-*g*-MPEG)

The graft block copolymer PAzoMA-*b*-(PELG-*g*-MPEG) was synthesized by click chemistry according to the literature.^48^ Typically, PAzoMA_59_-*b*-PAELG_50_ (0.35 g, 0.53 mmol of azido groups), alkyne-MPEG (1.86 g, 5.3 mmol), CuBr (0.76 g, 5.3 mmol) and PMDETA (0.89 g, 5.3 mmol) were dissolved in 10 mL DMF at a Schlenk tube. After three freeze–pump–thraw cycles, the tube was sealed under vacuum and immersed in a thermostatic oil bath at 40 °C for 72 h. The solution was diluted with THF and passed through a column of neutral oxide aluminum to remove copper salt. The product was obtained by precipitating into cold methanol, purified by reprecipitating three times from THF to cold diethyl ether and dried under vacuum overnight at 40 °C.

Yield: 87.5%, *M*_n_ (GPC) = 12.5 × 10^3^ kg mol^−1^, *M*_w_/*M*_n_ = 1.31, ^1^H NMR (CDCl_3_ + 15% TFA, *δ*/ppm): 7.80 (br, –C_6_H_4_–), 6.92 (br, –C_6_H_4_–), 4.62 (br, –CHCO–), 3.92 (br, –OCH_2_–), 3.80 (br, CH_3_O–), 3.63–3.54 (m, –OCH_2_CH_2_–), 3.37 (br, CH_3_O–), 1.05 (br, –CCH_2_–), 0.89 (br, CH_3_C–). The obtained polymer was denoted as PAzoMA_59_-*b*-(PELG_50_-*g*-MPEG).

### Preparation of self-assembly samples

PAzoMA_59_-*b*-(PELG_50_-*g*-MPEG) was dissolved in THF at 0.3 mg mL^−1^ and filtered through an N6 filter (0.45 μm) for the preparation of self-assembly samples and the measurement of the photo-responsive behavior. The self-assembled aggregate solution was prepared by adding the selective solvent water at the speed of 80 μL min^−1^. The volume ratio of the polymer solution to the selective solvent water was 5 : 5.

### Characterization

The molecular weights and polydispersity (*M*_w_/*M*_n_; *M*_w_: weight-average molecular weight, *M*_n_: number-average molecular weight) were determined by a gel permeation chromatograph (GPC) equipped with two Mixed-B columns (Polymer Laboratory, pore size = 10 μm; column size = 300 × 7.5 mm) and a refractive index detector (PerkinElmer Series 200) using DMF (0.01 mol L^−1^ LiBr) as the eluent at 40 °C with a flow rate of 1.0 mL min^−1^. The column system was calibrated by a set of monodispersed standard PMMA. ^1^H NMR spectra were recorded on a 500 Bruker NMR instrument using CDCl_3_ as the solvent and TMS as a reference standard for chemical shifts. Fourier transform infrared (FT-IR) spectra were recorded on a PerkinElmer Spectrum One spectrometer at frequencies ranging from 400 to 4000 cm^−1^ and samples were prepared into pellets by using KBr. The scanning electron microscopy measurement was performed using a field emission microscope (S-4800, HITACHI) with an accelerating voltage of 15.0 kV. The sample for SEM was prepared by drop-casting on drop (∼10 μL) of the self-assembled aggregate solution onto a clear silicon wafer and sputtered by Au before the measurement. The transmission electron microscopy (TEM) observation was carried out on a JEM-2100F JEOL TEM microscope with an accelerating voltage of 200 kV and one drop of the self-assembled solution was placed on a carbon-coated copper grid at the ambient temperature. The optical microscopy (Leica Dmlp, TMS94) equipped with a Sony digital camera was used to directly observe the aggregation morphologies and their images in the field of view of the optical microscopy were recorded with the computer in synchronization. A high-intensity lamp (Uvata UP115) was used to trigger the photo-isomerization of the azo chromophores by using 365 nm or 450 nm irradiation at 110 mW cm^−2^ with different irradiation times and the UV-vis spectra of the samples were measured with a Jasco V-50 spectrophotometer after irradiation.

## Results and discussion

### Synthesis of PAzoMA-*b*-(PELG-*g*-MPEG)

**Fig. 1:**
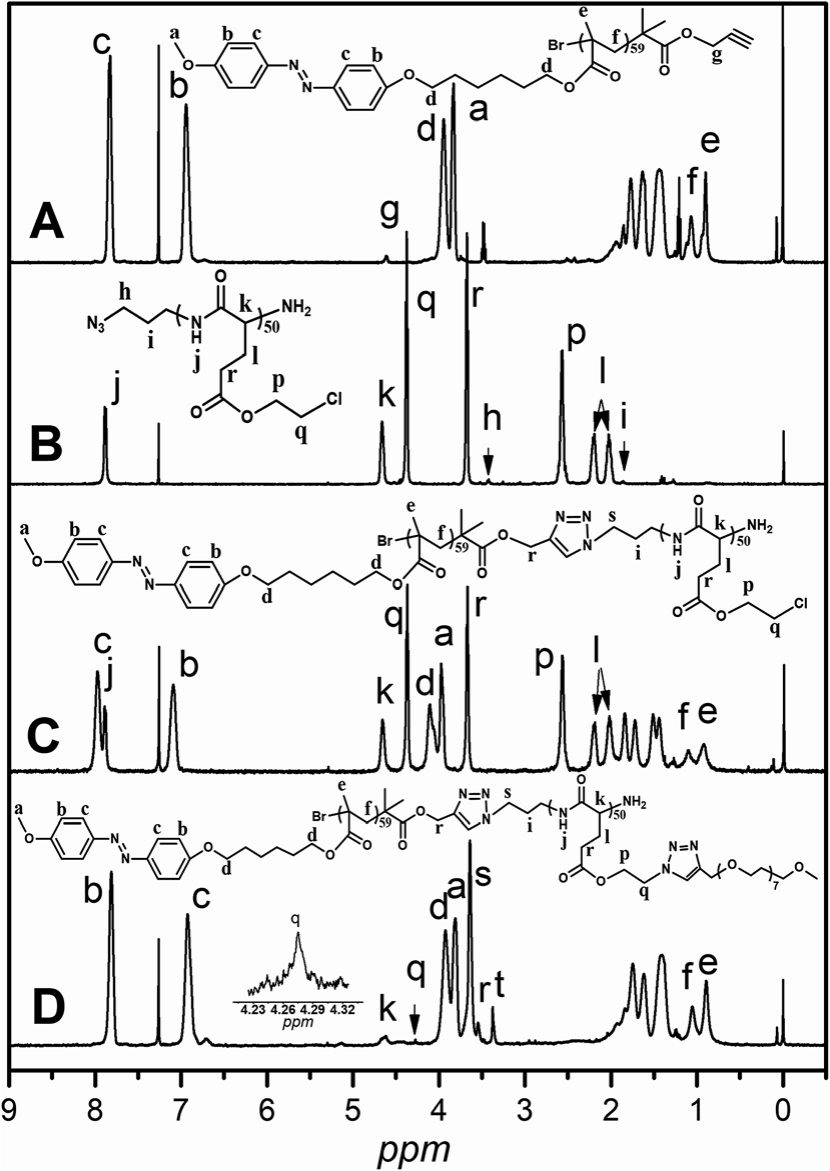
^1^H NMR spectra of alkyne-PAzoMA_59_ (A), azido-PCELG_50_ (B), PAzoMA_59_-*b*-PCELG_50_ (C) and PAzoMA_59_-*b*-(PELG_50_-*g*-MPEG) (D) in CDCl_3_ + 15% TFA.

**Fig. 2:**
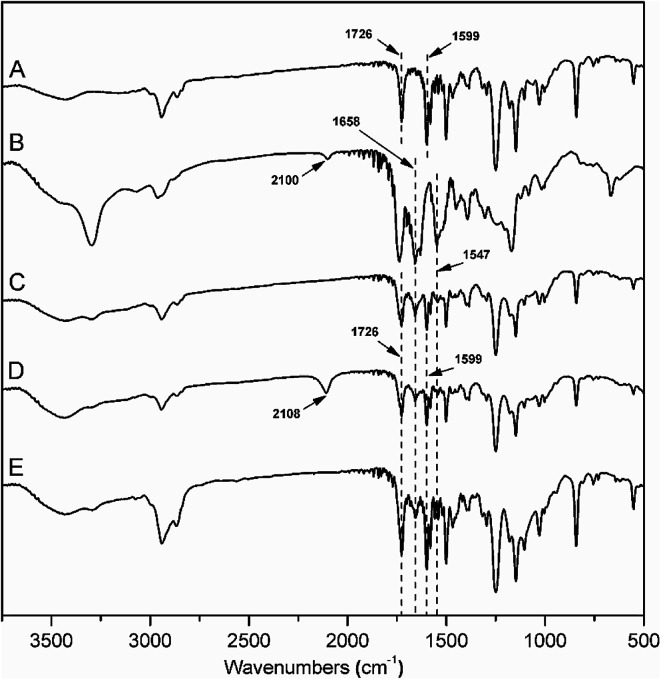
FT-IR spectra of alkyne-PAzoMA_59_ (A), azido-PCELG_50_ (B), PAzoMA_59_-*b*-PCELG_50_ (C), PAzoMA_59_-*b*-PAELG_50_ (D) and PAzoMA_59_-*b*-(PELG_50_-*g*-MPEG) (E).

As shown in Scheme 1, the graft block copolymer PAzoMA-*b*-(PELG-*g*-MPEG) was synthesized by the combination of ATRP, ROP and click reaction. The chemical structures of the synthetic polymers were characterized by ^1^H NMR, IR and GPC. Fig. 1 depicts the ^1^H NMR spectra of PAzoMA_59_-*b*-(PELG_50_-*g*-MPEG) and the corresponding precursors. As shown in Fig. 1A, the peaks at 7.81, 6.93, 3.93, 3.82, 1.06 and 0.89 ppm are attributed to the protons of the phenyl moiety (Fig. 1A: c and b), methylene (Fig. 1A: d), methoxyl group (Fig. 1A: a) and methacrylate backbone (Fig. 1A: f and e), respectively. It can be also clearly observed that the resonance signal of the peak originate from methylene (HC≡CC***H***_2_–) related to the initiator at 4.61 ppm (Fig. 1A: g), which means the functional group alkyne is not affected during ATRP process.^7^ By controlling the molar ratios of monomer AzoMA to initiator, the precursor alkyne-PAzoMA with predetermined degrees of polymerization can be prepared. The actual degree of polymerization (DP) can be estimated on the basis of the ^1^H NMR measurement by comparing the integral ratio of protons from the methylene (HC≡CC***H***_2_–) at 4.61 ppm (Fig. 1A: g) to the phenyl moiety (Fig. 1A: c or b) at 7.81 or 6.93 ppm. The sample was synthesized and denoted as alkyne-PAzoMA_59_ (DP = 59). The number-average molecular weight (*M*_n_) and the corresponding molecular weight distribution (*M*_w_/*M*_n_) of the sample from GPC measurement were 11.0 × 10^3^ kg mol^−1^ and 1.26 (Table 1), respectively. The vibration frequencies of the ester groups and phenyl groups at 1726 and 1599 cm^−1^ (Fig. 2A), respectively, can be clearly observed in the FT-IR spectrum of alkyne-PAzoMA_59_. The obtained results from ^1^H NMR, FTIR and GPC demonstrated that the alkyne-PAzoMA_59_ precursor was successfully synthesized by ATRP.

**Table 1:** Characterization of the synthesized polymers

Polymer	*M*_n_ a (g mol^−1^)	*M*_w_/*M*_n_^a^	DP_n_^b^	*f*_PAzoMA_ b
Alkyne-PAzoMA_59_	11 000	1.26	59	100
Azido-PCELG_50_	5200	1.29	50	—
PAzoMA_59_-*b*-PCELG_50_	11 200	1.25	—	70.5
PAzoMA_59_-*b*-PAELG_50_	12 100	1.29	—	65.3
PAzoMA_59_-*b*-(PELG_50_-*g*-MPEG)	12 500	1.31	—	46.7

a Determined by GPC in DMF-LiBr (0.01 mol L^−1^) with calibrated PMMA standards at 40 °C.

b Determined by ^1^H NMR.

**Fig. 3:**
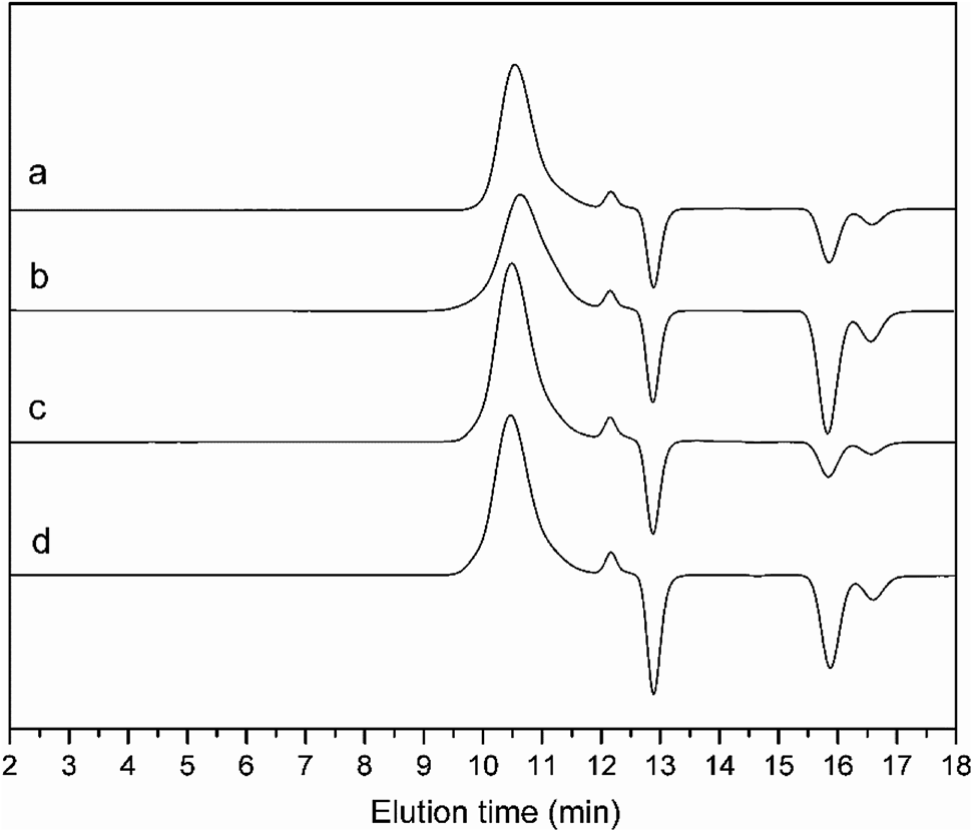
GPC curves of (a) alkyne-PAzoMA_59_, (b) azido-PCELG_50_, (c) PAzoMA_59_-*b*-PCELG_50_ and (d) PAzoMA_59_-*b*-(PELG_50_-*g*-MPEG).

To obtain azido functionalized PCELG precursor (*i.e.* azido-PCELG), 1-azido-3-aminopropane was used to initiate ROP of monomer CELG-NCA in DMF at room temperature.^7,54^^1^H NMR spectrum of azido-PCELG recorded in CDCl_3_ with TFA is shown in Fig. 1B. The resonance signals of the α-methylene (N_3_C***H***_2_CH_2_CH_2_–) and β-methylene protons (Fig. 1B: h and i) adjacent to the azido group originated from the initiator 1-azido-3-aminopropane can be observed at 3.42 and 1.84 ppm,^7^ respectively. The signals of protons of amide groups (Fig. 1B: j), α-methylene (Fig. 1B: r) and β-methylene (Fig. 1B: q) adjacent to chloro group, α-methine (Fig. 1B: k), β- and γ-methylene (Fig. 1B: l and p) originating from l-glutamic acid appear at 7.85, 3.67, 4.37, 4.65, 2.18–2.01 and 2.55 ppm, respectively. The degree of polymerization of the sample can be measured by ^1^H NMR and it value can be obtained by comparing the integral ratio of protons from the α-methylene (N_3_C***H***_2_CH_2_CH_2_–) protons (Fig. 1B: h) at 3.42 ppm to the α-methylene (Fig. 1B: r) adjacent to chloro group at 3.67 or the γ-methylene (Fig. 1B: p) originating from l-glutamic acid at 2.55 ppm. Therefore, the sample was denoted as azido-PCELG_50_. The number-average molecular weight (*M*_n_) and the corresponding molecular weight distribution (*M*_w_/*M*_n_) of azido-PCELG_50_ from GPC measurement (Fig. 3b) are 5.2 × 10^3^ kg mol^−1^ and 1.29 (Table 1), respectively. On the other hand, the characteristic absorption peak of the terminal azido group at 2100 cm^−1^ can be clearly observed in the FT-IR spectrum of azido-PCELG_50,_ as shown in Fig. 2B. At the same time, the characteristic absorption peaks of the amine I band and amine II band at 1658 and 1547 cm^−1^ (Fig. 2B), respectively, which relates to α-helix secondary structure of polypeptide.^56^ From the results of ^1^H NMR, GPC and FT-IR measurements, the azido-PCELG_50_ precursor was successfully synthesized.

The click reaction between the alkyne-PAzoMA_59_ and azido-PCELG_50_ precursors was performed to obtain the diblock copolymer PAzoMA_59_-*b*-PCELG_50_, as illustrated in Scheme 1. After click reaction, the GPC curve of block copolymer PAzoMA_59_-*b*-PCELG_50_ was unimodal and symmetrical, and shifted clearly toward the higher molecular weight region (Fig. 3 and Table 1). The chemical structures of diblock copolymer PAzoMA_59_-*b*-PCELG_50_ were further confirmed by ^1^H NMR and FT-IR measurements. ^1^H NMR and FT-IR spectra of PAzoMA_59_-*b*-PCELG_50_ are shown on Fig. 1C and 2C, respectively. The characteristic resonance signals of the peaks originating from PAzoMA block and PCELG block can be still clearly observed in ^1^H NMR spectrum of PAzoMA_59_-*b*-PCELG_50_ (Fig. 1C). At the same time, the absorption peak at 2100 cm^−1^ attributed to the terminal azido group of azido-PCELG_50_ disappeared at the FT-IR spectrum of PAzoMA_59_-*b*-PCELG_50_ (Fig. 2C) after click reaction. Besides the characteristic peaks at 1726 and 1599 cm^−1^ from PAzoMA_59_ block, FT-IR spectrum of PAzoMA_59_-*b*-PCELG_50_ clearly shows (Fig. 2C) the characteristic absorption peaks of the amine I band and amine II band at 1658 and 1547 cm^−1^ originating from PCELG_50_ block. Therefore, block copolymer PAzoMA_59_-*b*-PCELG_50_ was successfully synthesized. By ^1^H NMR measurements, the PAzoMA segment content in block copolymer PAzoMA_59_-*b*-PCELG_50_ was 70.5% (Table 1), which was consistent with the values calculated from the corresponding PAzoMA_59_ and PCELG_50_ precursors within the error. The obtained block copolymer PAzoMA_59_-*b*-PCELG_50_ was further modified by azide reaction between the chloro groups of PCELG block in block copolymer with NaN_3_ in DMF to prepare the block copolymer containing the polypeptide block with azido side groups, which was denoted as PAzoMA_59_-*b*-PAELG_50_. FT-IR spectrum of PAzoMA_59_-*b*-PAELG_50_ is shown on Fig. 2D. Besides the characteristic peaks at 1726 and 1599 cm^−1^ from Azo-polymer block, the amine I band and amine II band peaks at 1658 and 1547 cm^−1^ originating from polypeptide block, the characteristic absorption peak at 2108 cm^−1^ contributed to the azido side groups in polypeptide block, denoted as PAELG, can be clearly observed in the FT-IR spectrum of PAzoMA_59_-*b*-PAELG_50_ (Fig. 2D) in comparison with that of PAzoMA_59_-*b*-PCELG_50_ (Fig. 2C). Moreover, by GPC analysis, the symmetrical and unimodal elution peak of PAzoMA_59_-*b*-PAELG_50_ can be observed and the elution time of PAzoMA_59_-*b*-PAELG_50_ was almost same as that of PAzoMA_59_-*b*-PCELG_50_ (Table 1), indicating that molecular weight did not decrease after azidonation reaction. Graft block copolymer PAzoMA_59_-*b*-(PELG_50_-*g*-MPEG) was synthesized by click reactions between the PAzoMA_59_-*b*-PAELG_50_ containing azido side groups with the alkyne-terminated MPEG and their chemical structures were characterized by ^1^H NMR, FT-IR and GPC techniques. Fig. 1D shows the ^1^H NMR spectrum of the graft block copolymer PAzoMA_59_-*b*-(PELG_50_-*g*-MPEG). Besides the characteristic signals at 7.81 and 6.93 ppm (Fig. 1D: b and c) originating from PAzoMA blocks and those at 4.37 and 4.65 ppm (Fig. 1D: q and k) assigned to the protons of the polypeptide blocks can be also observed, these proton signals originated from MPEG segments at 3.47–3.75 ppm (Fig. 1D: s) assigned to –C**H**_2_C**H**_2_– and 3.38 ppm (Fig. 1D: t) attributed to methoxyl groups can be clearly observed. On the other hand, the absorption peak at 2108 cm^−1^ attributed to the azido side groups of PAzoMA_59_-*b*-PAELG_50_ (Fig. 2D) disappeared at the FT-IR spectrum of PAzoMA_59_-*b*-(PELG_50_-*g*-MPEG) (Fig. 2E) after click reaction. Besides the characteristic peaks at 1726 and 1599 cm^−1^ from PAzoMA_59_ block, FT-IR spectrum of PAzoMA_59_-*b*-(PELG_50_-*g*-MPEG) clearly shows (Fig. 2E) the characteristic absorption peaks of the amine I band and amine II band at 1658 and 1547 cm^−1^ originating from polypeptide block. At the same time, the result of GPC measurement also showed that the molecular weight of PAzoMA_59_-*b*-(PELG_50_-*g*-MPEG) was larger than that of the corresponding precursor PAzoMA_59_-*b*-PAELG_50_ (Fig. 3 and Table 1). Therefore, block copolymer PAzoMA_59_-*b*-(PELG_50_-*g*-MPEG) was successfully synthesized by the combination of ATRP, ROP and click reaction.

### Self-assembly behavior of graft block copolymer PAzoMA-*b*-(PELG-*g*-MPEG) in solution

**Fig. 4:**
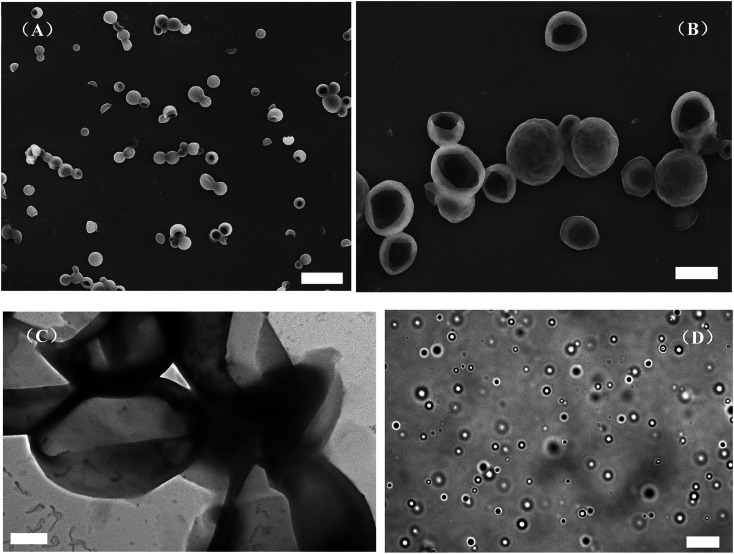
SEM images (A and B), TEM image (C) and optical micrograph (D) of the vesicles formed by PAzoMA_59_-*b*-(PELG_50_-*g*-MPEG) in THF with 0.3 mg mL^−1^ initial concentration through adding H_2_O. The scale bars represent 6.0 μm in (A), 2.0 μm in (B), 1.0 μm in (C) and 20.0 μm in (D).

The aggregate in water was prepared from PAzoMA_59_-*b*-(PELG_50_-*g*-MPEG) by adding water progressively into THF solution of the graft block copolymer and the volume ratio of the polymer solution to the selective solvent water was 5 : 5. The combined techniques of SEM, optical microscopy and TEM were used to explore the morphologies and structures of the aggregates. Fig. 4 shows the morphologies of PAzoMA_59_-*b*-(PELG_50_-*g*-MPEG) aggregates from the selective solvent H_2_O. PAzoMA_59_-*b*-(PELG_50_-*g*-MPEG) formed vesicles with an average diameter of *ca.* 1.83 μm based on the SEM results (Fig. 4A and B, see ESI, Fig. 1SA†), where a larger “cave” appeared in some aggregates (Fig. 4B, see ESI, Fig. 2S†), indicating the formed aggregates are vesicles. The vesicular structure is also proved by TEM results (Fig. 4C). The hydrophobic PAzoMA blocks in the graft block copolymers formed the wall of vesicles due to the hydrophobic interaction. The formation of the aggregate structures might be as follows:^26,57,58^ the self-assembly occurs when the content of the addition water reaches a critical point. With more water addition to the medium, THF in the interior of the aggregates diffuses out of cavities faster than the rate at which water diffuses in, where a different hydrostatic pressure can be created with the lower pressure on the inside. Thus, the thinnest part of the wall may be break and the opening in the aggregates can be formed. To further confirm the aggregate structure, the optical microscopy was employed for the structure detection. The optical image of the resulting aggregate solution is shown in Fig. 4D. It appears that the graft block copolymer can self-assemble into a well-defined vesicular structure with the average diameter of *ca.* 2.80 μm (see ESI, Fig. 1SB†). On the other hand, some micelle-like structure can be observed in the optical image (Fig. 4D). This is due to the Brownian movement of the aggregates in solution, which causes the aggregates to be in different focal planes. The video recording of the aggregate movement in solution can explain this phenomenon very well (see ESI, Video 1†). Compared with the giant vesicles prepared from lipids,^59^ hyperbranched copolymer^60–62^ and block copolypeptide,^40,41,63^*et al.*, we report for the first time the micrometer-size vesicles formed by graft block azobenzene-containing copolymer with polypeptide block containing two kinds of different rod-like polymer chains derived from different interactions (π–π stacking interaction and intramolecular hydrogen bonding), which may render the giant vesicles advanced functions.

### Photo-responsive transition behavior

**Fig. 5:**
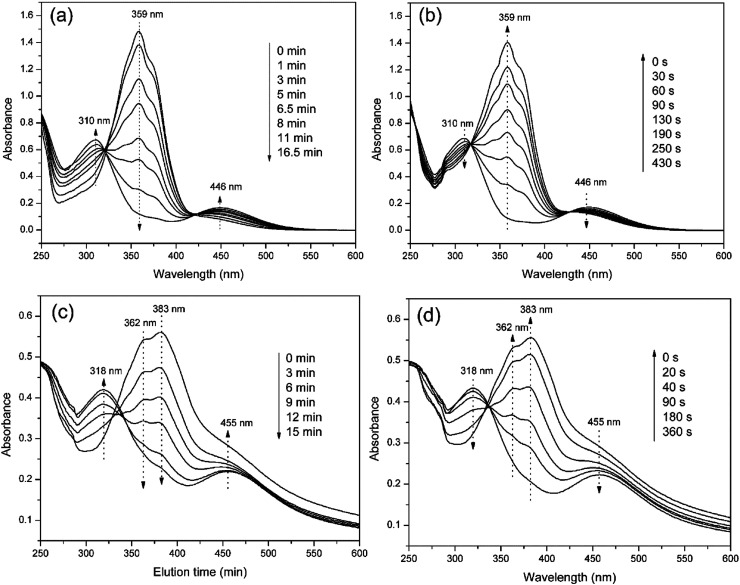
UV-vis absorption spectra of PAzoMA_59_-*b*-(PELG_50_-*g*-MPEG) in solution (a and b) and in aggregate (c and d).

The photo-responses of PAzoMA_59_-*b*-(PELG_50_-*g*-MPEG) in THF solutions and in aggregate solutions were evaluated by UV-vis spectroscopy. Fig. 5 shows UV-vis spectra of PAzoMA_59_-*b*-(PELG_50_-*g*-MPEG) in THF solution and in aggregate solution irradiated with UV light at 365 nm or vis light at 450 nm for different times until the photostationary state reached. From Fig. 5a, it is clearly observed that the peak absorbance at 359 nm attributed to the π–π* transition of *trans*-azo isomers decreases gradually with UV irradiation. At the same time, an increase in the absorbance at 446 nm assigned to the n–π* transition of *cis*-azo isomers occurs. Moreover, when the isomerization continues, more and more *cis* isomers are generated and an increment of the absorbance at 310 nm belonged to the π–π* transition of *cis*-azo isomers appears.^26,36,37,64^ At last, the wavelength of maximum absorption gradually shifts from 359 nm to 310 nm. The results indicates the *trans*-to-*cis* isomerization of azobenzene chromophores. On the other hand, UV-vis spectra can start to recover the initial shape under vis light irradiation at 450 nm with increasing the irradiation times (Fig. 5b).

**Scheme 2:**
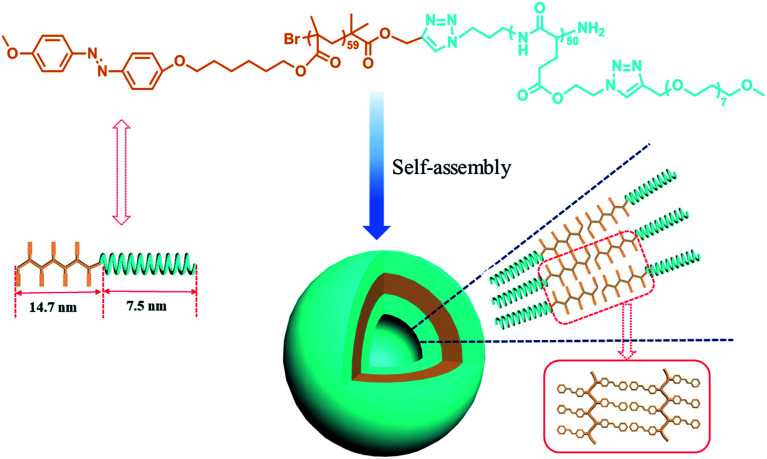
Schematic representation of the amphiphilic graft block copolymer PAzoMA_59_-*b*-(PELG_50_-*g*-MPEG) and the vesicle structure.

When PAzoMA_59_-*b*-(PELG_50_-*g*-MPEG) in THF solution self-assembled into vesicles, the isomerization behavior of azobenzene chromophores was different to that in solution. In comparison with the spectra of PAzoMA_59_-*b*-(PELG_50_-*g*-MPEG) in THF solution, the spectra of vesicle solution evidences broadening and bathochromic shifting of the π–π* band (Fig. 5c and d). From Fig. 5c, when prior to UV irradiation of 365 nm light, the n–π* transition of *cis*-azo isomers almost can not be observed and it is easily observed that the maximum absorption wavelength of the π–π* transition of *trans*-azo isomers is shifted from 359 nm in THF solution to 383 nm in aggregation solution, which indicates the predominant formation of azobenzene J-aggregates.^26,36^ Under the successive irradiation of 365 nm UV light, the absorption strengths of the peak at 383 nm for azobenzene J-aggregates and another shoulder peak at 362 nm for azobenzene non-associations successively decrease in the vesicle solution. At the same time, an increment of the absorbance at 318 nm belonged to the π–π* transition of *cis*-azo isomers appears and the absorbance at 455 nm related to the n–π* transition of *cis*-azo isomers can be clearly observed (Fig. 5c). The results indicates the occurrence of the *trans*-to-*cis* isomerization of azobenzene chromophores in the vesicles. Furthermore, the photo-isomerization rate of the vesicle solution is slower than that of the corresponding THF solution (see ESI, Fig. 3S†). The rate values for PAzoMA_59_-*b*-(PELG_50_-*g*-MPEG) in the THF solution and the vesicle solution are 2.45 × 10^−3^ s^−1^ and 2.03 × 10^−3^ s^−1^ (see ESI, Fig. 3S†), respectively. The obtained results verify that the azobenzene chromophores are more orderly arranged in the vesicles than in the THF solution. Generally speaking, azobenzene H-aggregate is more stable than azobenzene J-aggregate, which is due to the more compact packing of the azobenzene segments in H-aggregates. However, azobenzene J-aggregates in the vesicle solution are only observed in our research system. The probable reason for this as follow: the shells of vesicles are formed by hydrophilic polypeptides with rigid α-helical structures (Scheme 2),^45,65^ which can provide more free volumes and reduce the chain repulsion interactions in the shells, and this is beneficial to the formation of vesicular wall by hydrophobic PAzoMA chains according to the looser J-aggregate behavior. The PAzoMA chain in PAzoMA_59_-*b*-(PELG_50_-*g*-MPEG) was calculated to be 14.7 nm if it adopts a rigid conformation.^66^ On the other hand, the length of the polypeptide chain in rigid α-helical conformation was calculated to be 7.5 nm.^45,56^ The average vesicle-wall thickness obtained from TEM determination was approximately 42.5 nm (see ESI, Fig. 4S†). Therefore, the graft block copolymer PAzoMA_59_-*b*-(PELG_50_-*g*-MPEG) is thought to pack in a head-to-head arrangement to form a bilayer structure in vesicles (Scheme 2), in which the hydrophobic PAzoMA chains with π-conjugated azo chromophores in J-aggregate behavior were packed in the middle layer wall and the hydrophilic polypeptide chains in α-helical conformation were packed in corona shell of vesicles.^40,63^

On the other hand, the successive irradiations of 450 nm light on the vesicle solution can recover the initial shape (Fig. 5d), indicating that the *cis*-to-*trans* isomerization of azobenzene chromophores easily occurs due to larger free volume in the wall of the vesicles.

## Conclusion

Novel amphiphilic graft block azobenzene-containing copolymer PAzoMA_59_-*b*-(PELG_50_-*g*-MPEG) was successfully synthesized *via* a combination of ATRP, ring-opening polymerization and click reaction. The chemical structures were characterized in detail by ^1^H NMR, IR and GPC. The amphiphilic graft block azobenzene-containing copolymer in selective solvent H_2_O self-assembled into the micrometer-scale vesicles. The investigation of the photo-isomerization behavior of PAzoMA_59_-*b*-(PELG_50_-*g*-MPEG) in solution and in vesicular solution showed *trans*-to-*cis* isomerization in solution was quicker than that in vesicular solution and azobenzene J-aggregates in the vesicle solution were only observed. The formation mechanisms were explored and it was beneficial to the formation of the giant vesicles due to the arrangement of the hydrophilic polypeptides with the rigid in α-helical conformation on the inner and outer surfaces of vesicles. The research results enriched the field about self-assembly of amphiphilic block copolymers containing different rod-like polymer chains derived from different interactions.

## Author contributions

The manuscript was written through contributions of all authors. All authors have given approval to the final version of the manuscript. Xiaohua He designed research; Chunyan Gao performed the synthesis of graft block copolymers; Jianxiang Wu only performed the self-assembly and photoresponsive property of graft block copolymer; Jianxiang Wu and Chunyan Gao contributed equally to the work.

## Conflicts of interest

There are no conflicts of interest to declare.

## Supplementary Material

RA-010-C9RA10351A-s001

RA-010-C9RA10351A-s002
