# Supplementary material for: Novel amphiphilic graft block azobenzene-containing copolymer with polypeptide block: synthesis, self-assembly and photo-responsive behavior
Source: RSC Adv. 2020 Feb 5;10(10):5747–57. doi: 10.1039/c9ra10351a (PMC9049285; doi:10.1039/c9ra10351a)
Supplement: RA-010-C9RA10351A-s001 [file RA-010-C9RA10351A-s001.pdf]

Electronic Supplementary Material (ESI) for RSC Advances.
This journal is © The Royal Society of Chemistry 2020

Electronic Supplementary information (ESI)

Novel amphiphilic graft block azo-copolymer with polypeptide block: synthesis, self-assembly and photo-responsive behaviour

Xiaohua He,* Jianxiang Wu, Chunyan Gao

***Corresponding author: e-mail: xhhe@chem.ecnu.edu.cn (X. He)**

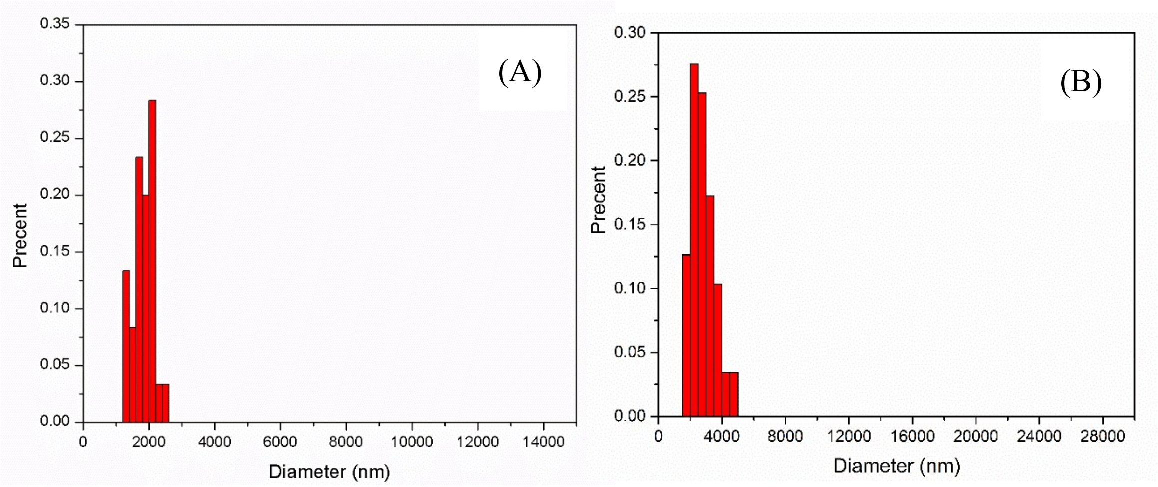

Figure 1S Size distribution of the giant vesicles: (A) SEM and (B) optical microscopy.

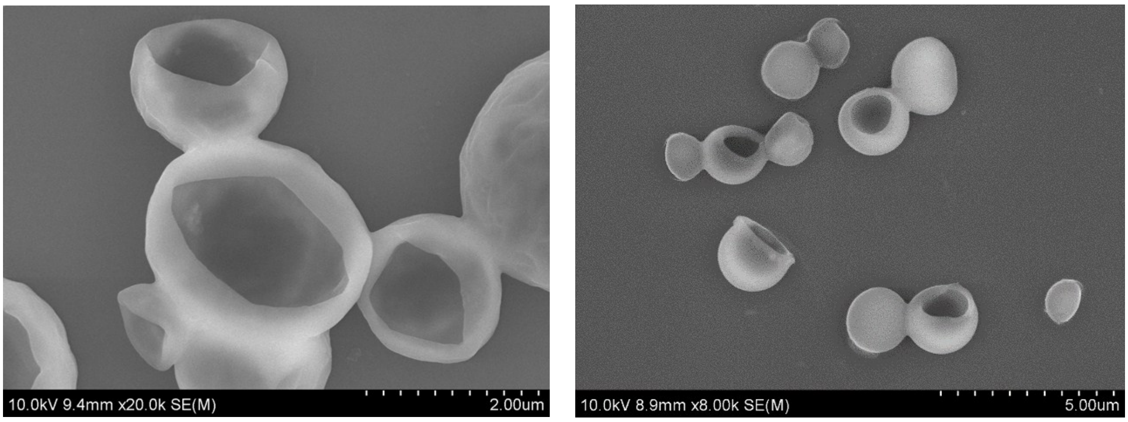

Figure 2S SEM images of the vesicles formed by PAzoMA$_{59}$-*b*-(PELG$_{50}$-*g*-MPEG) in THF with 0.3 mg/mL initial concentration through adding the selective solvent H_2O.

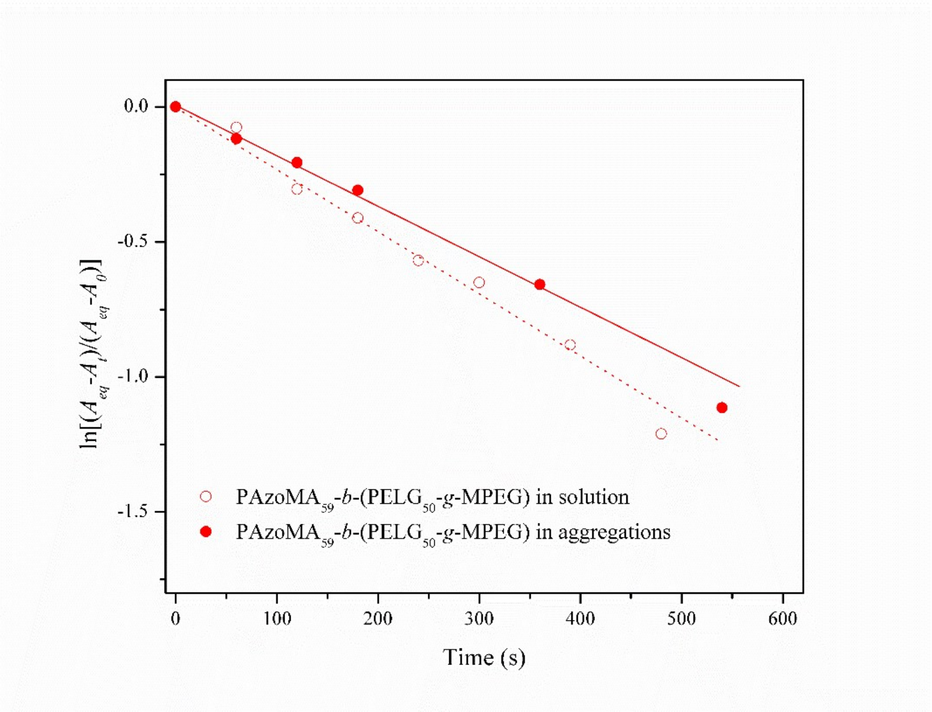

Figure 3S Plots of the first-order *trans*-to-*cis* photoisomerization of PAzoMA$_{59}$-*b*-(PELG$_{50}$-*g*-MPEG) in THF solution and in vesicle solution.

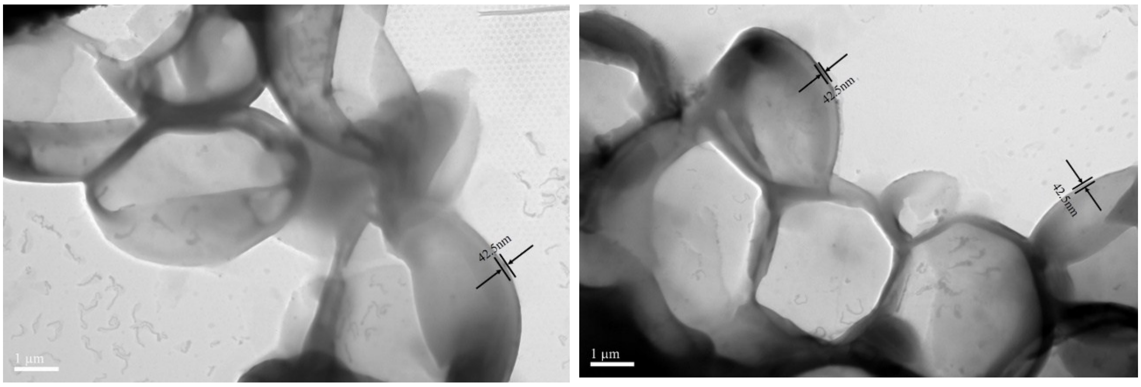

Figure 4S TEM images of the vesicles formed by PAzoMA$_{59}$-*b*-(PELG$_{50}$-*g*-MPEG) in THF with 0.3 mg/mL initial concentration through adding the selective solvent H_2O.

Video 1. The video recording of the aggregate movement in solution
